# Ectoparasitic prevention compliance in dogs based on prescription records from veterinary practices in Poland

**DOI:** 10.1186/s13071-026-07374-5

**Published:** 2026-07-22

**Authors:** Michał Ceregrzyn, Hina Naz, Robert P. Lavan, Rob Armstrong

**Affiliations:** 1 MSD Animal Health Poland, Chłodna 51, 00-867 Warsaw, Poland; 2 Celeritas Digital, Karachi, Pakistan; 3 Merck Animal Health, 126 E. Lincoln Ave, Rahway, NJ 07065 USA

**Keywords:** Ectoparasiticide, Fleas, Ticks, Dogs, Isoxazoline, Medication adherence, Poland

## Abstract

**Background:**

Fleas and ticks are common and harmful ectoparasites in dogs. They feed on blood, cause irritation, and transmit various pathogens. In Poland, the primary species *Ctenocephalides felis* (fleas), *Ixodes ricinus*, and *Dermacentor reticulatus* (ticks) pose a year-round threat. Veterinary organizations recommend continuous year-round protection against ectoparasites. However, actual owner compliance with this recommendation remains unknown.

**Methods:**

This retrospective study analyzed medical records from 2021 to 2023 across 45 veterinary practices in Poland. A total of 44,140 ectoparasiticide prescriptions were reviewed for 19,351 unique dogs. Products were categorized by active ingredient and duration of efficacy into four groups: 12-week isoxazolines, monthly isoxazolines, non-isoxazoline products, and collars. Protection coverage was estimated based on the number of doses prescribed and the labeled duration of each product, assuming all medications were administered on time, starting on the date of purchase.

**Results:**

The mean duration of protection against ticks over 3 years was 42.0 weeks for collars, 27.5 weeks for 12-week isoxazolines, 14.2 weeks for monthly isoxazolines, and 6.7 weeks for non-isoxazoline products. All durations were significantly different. Overall, the average protection provided to all dogs in the study was only 22.5% of the recommended year-round coverage. Most dogs received only one dose per year. Only 9.1% of dogs received at least one dose annually for three consecutive years, and just 0.1% received complete (> 150 weeks) year-round protection for all 3 years.

**Conclusions:**

These findings indicate that compliance with year-round ectoparasitic protection in Poland is significantly below recommended levels. Most dogs are not adequately protected throughout the year, and very few receive consistent protection over multiple years. This leaves a substantial portion of the canine population vulnerable to ectoparasitic infestations and vector-borne diseases. Veterinary clinics must adopt objective compliance tracking and proactively develop and refine communication strategies to enhance adherence to parasite prevention protocols and minimize the risk of parasite-related health issues in dogs, and consider the role of longer-acting parasiticides to improve adherence.

**Graphical Abstract:**

**Figure:**
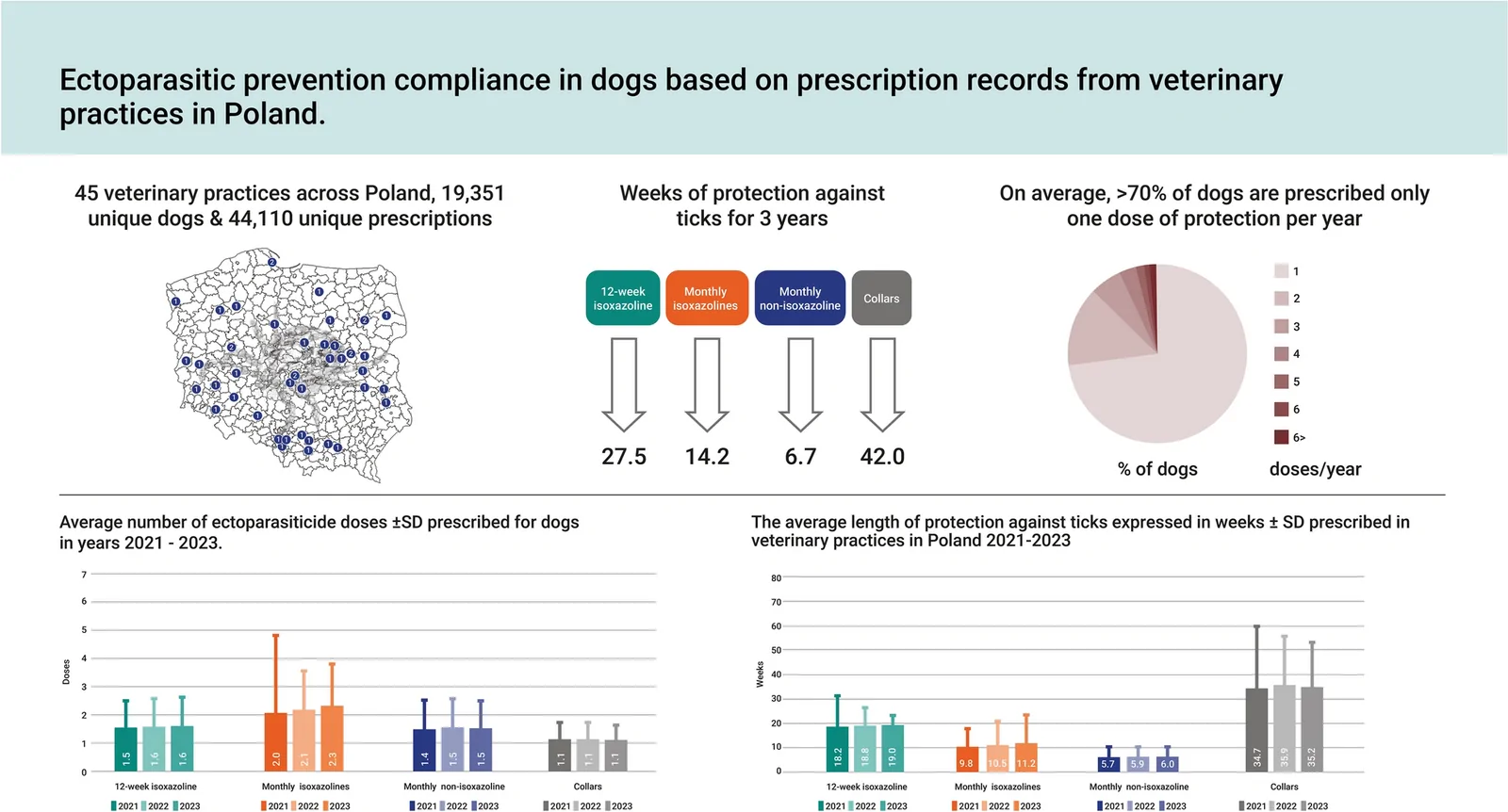

**Supplementary Information:**

The online version contains supplementary material available at https://doi.org/10.1186/s13071-026-07374-5.

## Background

Ticks and fleas are among the most prevalent ectoparasites infesting dogs in Europe [1]. These parasites pose a year-round threat to pets [1–5]. Documented year-round ectoparasite threats support veterinary recommendations for 12-month ectoparasite control for dogs [1, 6]. Flea infestation prevalence on dogs is high [7], and the most common flea species is *Ctenocephalides felis* [8, 9]. However, in Poland, the primary ectoparasite threats for domestic dogs are ticks, including *Ixodes ricinus*, *Ixodes crenulatus*, *Ixodes hexagonus*, *Ixodes rugicollis*, and *Dermacentor reticulatus* [2, 3, 10].

Protection against vector-borne diseases is the most crucial reason for using ectoparasiticides, and it requires highly effective flea and tick control. Fleas are vectors of infectious agents, including *Rickettsia* spp. and *Anaplasma spp.* [11, 12]. In Europe, the prevalence of tick-borne disease (TBD) in companion animals, particularly dogs, has risen significantly in recent decades [13]. Babesiosis, borreliosis, and anaplasmosis are the most prominent TBDs for dogs in Europe and Poland [13–16]. *Borrelia burgdorferi* and the tick-borne encephalitis virus are also dangerous zoonotic pathogens [17]. Significant risk factors for babesiosis—and likely other TBDs—in dogs include living in rural areas, kennels, or animal shelters; inhabiting an endemic region; season (associated with increased tick activity); and lack of acaricide treatment [18]. Year-round protection against fleas is essential for dogs suffering from flea-allergic dermatitis because flea bite hypersensitivity is a common cause of skin disease in dogs [19, 20].

The risk of ectoparasite infestations is likely to persist or even increase because wild animals serve as reservoirs for ticks and tick-borne pathogens [21, 22]. For example, *Babesia canis* was detected in the blood of 18% of wolves in Poland, while *Anaplasma phagocytophilum* was found in 2% of tested wolves [23]. Additionally*, A. phagocytophilum* DNA was identified in 5.8% of various wild carnivores, including red foxes [24]. Since populations of red foxes [25] and wolves [26] are increasing in Poland, the risk of ectoparasite infestations and vector-borne diseases for domestic dogs is likely to remain high, underscoring the need for adequate and continuous protection of pets.

In Poland, dogs may be prescribed various pharmaceutical products for ectoparasite protection, with effectiveness lasting between 1 and 7 months [25]. Studies to date indicate that a minimal number of dogs in other countries receive protection for a whole year, as recommended by veterinary guidelines and veterinarians [25–29]. Despite the recommendations, actual compliance tends to be low, with protection periods shorter than advised [25, 27]. Since the last study on ectoparasitic compliance was conducted a few years ago [25], this paper examines the prescribing patterns of ectoparasiticides by veterinarians for dogs in Poland from 2021 to 2023.

## Methods

Medical records documenting tick and flea medication (ectoparasiticide) prescriptions were obtained from 45 veterinary practices across Poland through the practice management system Klinika XP (BRI sp. z o.o, https://www.klinikaxp.pl/), with the consent of each participating veterinary practice. All records had the pet owner and pet identities concealed before accession. Only the postal codes of veterinary practices that submitted data were available. All records for 2021–2023 were collected, and only records pertaining to dogs were included, with no restriction on age, gender, neutering status, body weight, or geographical location within Poland. Transaction reports generated by practice software included itemized records with a unique veterinary practice code, postal area code, unique animal code, prescription date, species, breed, dog birth date, weight, medicine name, transaction date, and quantity of prescribed medication.

Prescribed medications were identified and categorized based on their summaries of product characteristics (SPC) listed in the Register of Medicinal Products Authorized for Marketing in Poland or the European Medicines Agency register. Only products indicated for treating or preventing ectoparasites, including endectocide combination products, were included in the analysis.

Systematic filtering steps were applied to database records to ensure data quality and relevance. Records pertaining to species other than dogs, entries with zero or blank values in the quantity column, records exceeding reasonable expected quantity thresholds, medication prescriptions classified as exclusively endoparasiticides, unrecognized classifications and mismatches between species and prescribed products, and multiple same-day purchases of various products under one animal identity number were not included in the final database. To avoid including potential excessive prescribing that was not assigned to the animal in the database, an additional filter was applied based on product-specific quantity—the recommended number of doses over 3 years. Records of dogs prescribed various product categories during the study period, indicating a switch in product category, were excluded from the final database. The record filtering provided a high-quality dataset suitable for analysis, reducing the dataset from 101,475 to 44,140 prescription records and the animal count from 38,923 to 19,351 unique dogs.

Two critical assumptions for calculating medication adherence from these records are that all medication prescriptions recorded were administered according to the timing and dosage specified in each product’s SPC, and that all ectoparasiticide doses prescribed were administered to the animal stated in the medical record, starting at or near the date of purchase.

**Fig. 1:**
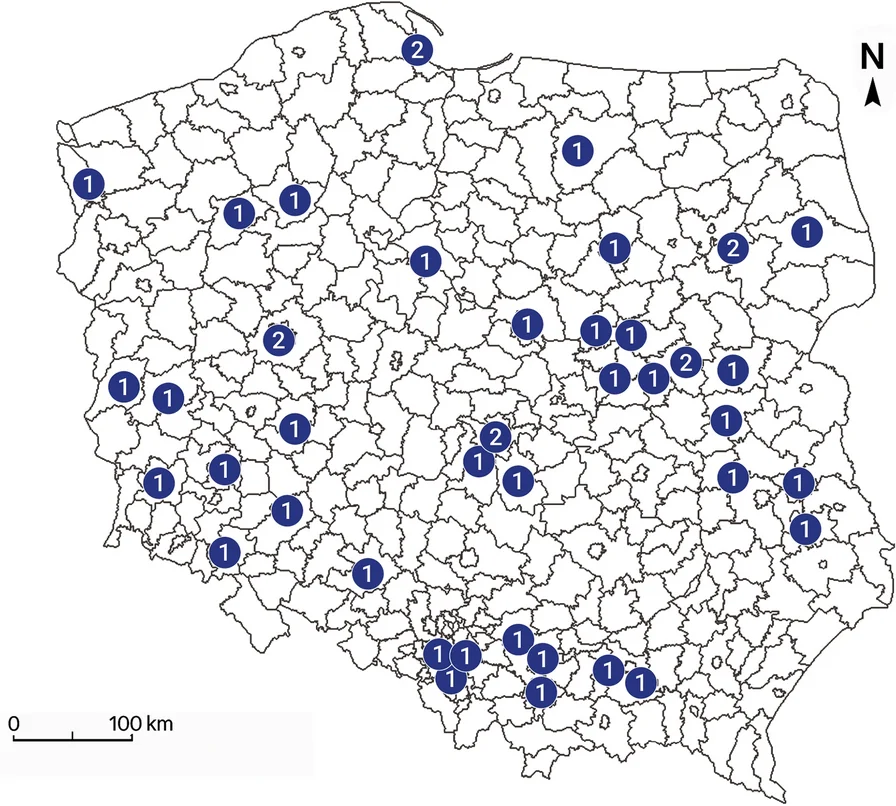
Approximate locations of 45 participating veterinary practices in Poland. Each circle represents a practice participating in the study

The postal codes of 45 participating veterinary practices were mapped to show their geographical distribution in Poland (Fig. 1). One-third of the practices were in a large triangular region, including the Warsaw metropolitan area, Lublin, and Łódź, while the remaining practices were distributed across Poland.

## Results

### Dog population description

The final database included 19,351 unique dogs and 44,140 transaction records, representing all dogs prescribed at least one dose of ectoparasitic treatment at participating veterinary hospitals between 2021 and 2023.

**Fig. 2:**
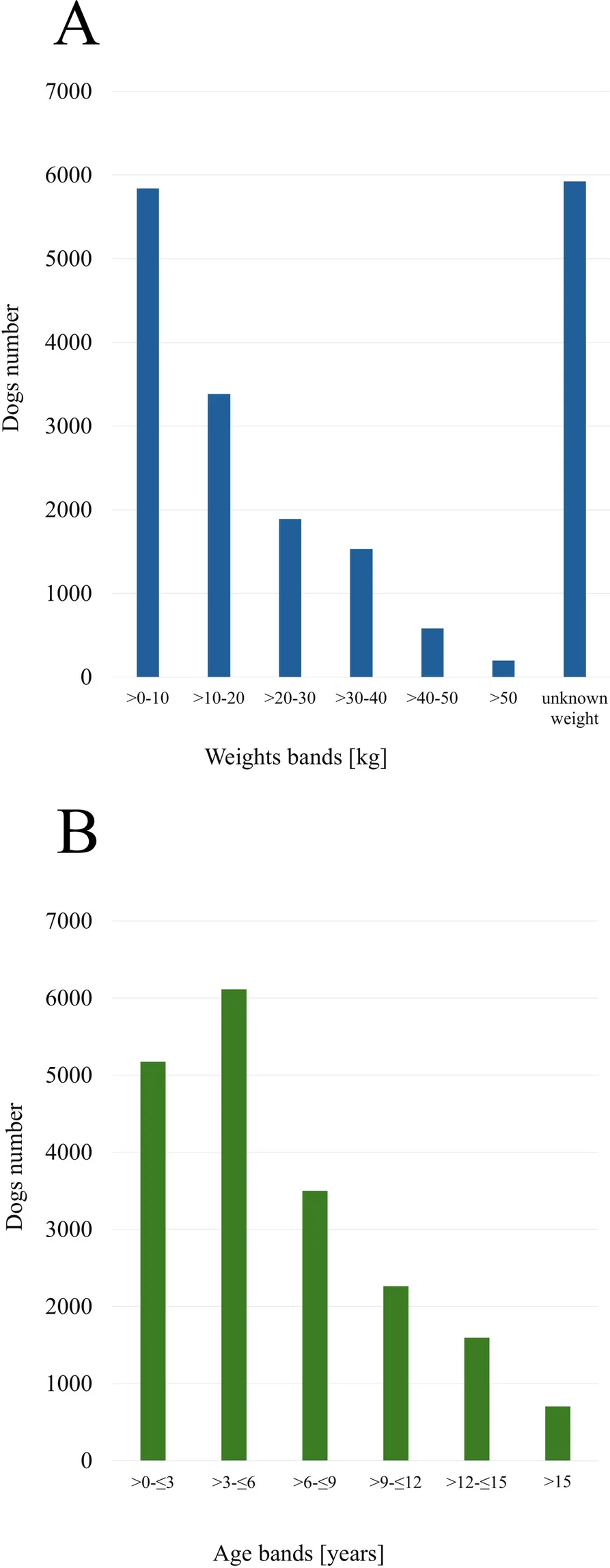
Weight (**A**) and age (**B**) distribution of 19,351 dogs prescribed an ectoparasiticide in 45 veterinary practices in Poland in 2021–2023. The dogs’ age was calculated as of the study end date, December 31, 2023

From the total pool of dogs, body weight data were available for 13,428. A large group of 5915 dogs (30.6% of 19,351 dogs) had no recorded weight. Additionally, eight dogs with recorded weights above 110 kg were excluded from body weight analysis due to suspected data entry errors. Therefore, the weight of the dogs included in the analysis ranged from 1 to 110 kg, with a mean of 16.9 kg. Smaller dogs, weighing between 1 and 10 kg, predominated, comprising 5839 dogs (43%), while larger dogs weighing over 50 kg accounted for 5% of the analyzed population (Fig. 2).

Each dog’s age was calculated as of the study end date (December 31, 2023) using its recorded birth date. Five dogs with calculated age greater than 24 years and two dogs with age less than 1 month at the study end date were excluded from the age distribution analysis due to suspected data entry errors. Therefore, 19,344 dogs were analyzed. Dogs’ ages ranged from 2 months to 24 years, with a mean age of 6.1 years. The largest age group (32%) consisted of dogs aged 3–6 years, while dogs aged 15 years and above accounted for 4% of the analyzed population (Fig. 2).

Breed classifications were not analyzed because participating veterinary practices did not use a standardized breed identification system.

### Tick and flea medication prescriptions

**Fig. 3:**
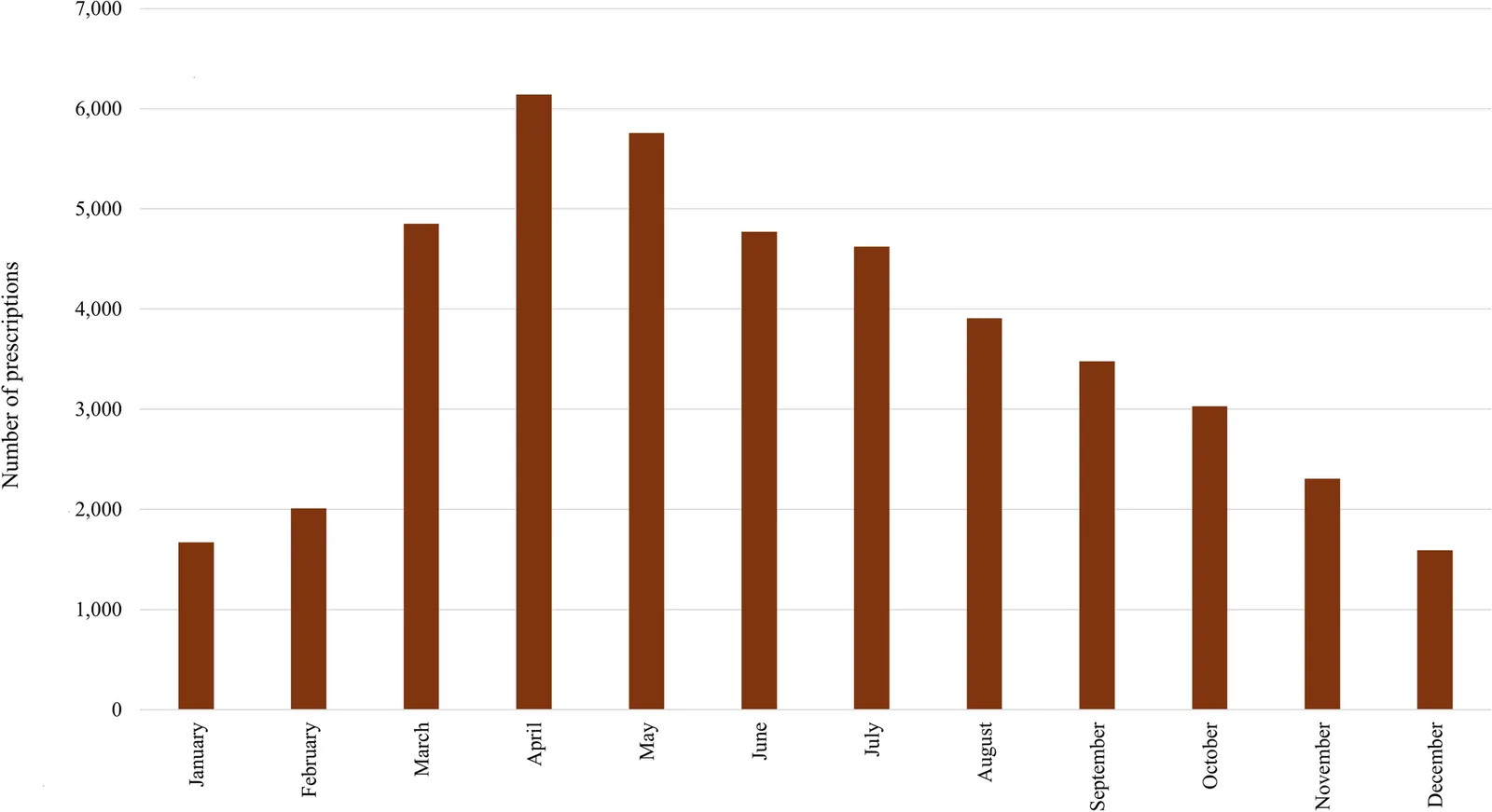
Number of prescriptions for ectoparasiticides each month for 19,351 dogs in 45 veterinary practices in Poland, 2021–2023

Prescribing patterns show seasonal variation throughout the analysis period (Fig. 3). The highest number of transactions were recorded in April, and the lowest in January. From the spring peak of prescriptions, the number gradually declined throughout the year.

### Doses prescribed for dogs

The average number of doses per product category per year (Table 1) shows that prescribing of isoxazolines increased significantly from 2021 to 2023, while prescribing of non-isoxazolines and collars remained unchanged (with no statistical significance).

**Table 1:** The average number of ectoparasiticide doses ± standard deviation (SD) prescribed for dogs in 2021, 2022, and 2023

Product category	2021	2022	2023
12-week isoxazoline	1.52 ± 0.90^b,c,d^	1.56 ± 0.91 (+3%)^b,d^	1.58 ± 0.89 (+1%)^*,b,c,d^
Monthly isoxazoline	1.99 ± 1.71^a,c,d^	2.14 ± 1.91 (+8%)*^,a,c,d^	2.29 ± 2.05 (+7%)^*,**,a,c,d^
Monthly non-isoxazoline	1.43 ± 1.13^a,b,d^	1.48 ± 1.12 (+3%)^b,d^	1.48 ± 1.17 (0%)^a,b,d^
Collar	1.08 ± 0.33^a,b,c^	1.10 ± 0.38 (+2%)^a,b,c^	1.07 ± 0.34 (−3%)^a,b,c^

Relative changes in 2022 and 2023 versus the prior year are shown in brackets

Significance of differences among years within product categories

^*^Significantly different from 2021 (unpaired *t*-test, *P* < 0.05)

^**^Significantly different from 2022 (unpaired *t*-test, *P* < 0.05)

Significance of differences among product categories within years

^a^Significantly different from 12-week isoxazolines (unpaired *t*-test, *P* < 0.05)

^b^Significantly different from monthly isoxazolines (unpaired *t*-test, *P* < 0.05)

^c^Significantly different from monthly non-isoxazolines (unpaired *t*-test, *P* < 0.05)

^d^Significantly different from collars (unpaired *t*-test, *P* < 0.05)

Most dogs received one dose (treatment) per year in each product category. More than 90% of dogs were prescribed one dose (collar) annually; 77.2%, 76.0%, and 75.8% of dogs were prescribed one dose per year of monthly non-isoxazolines in 2021, 2022, and 2023, respectively. For the 12-week isoxazolines, this proportion also decreased from 66.8% in 2021 to 62.9% in 2023. For monthly isoxazolines, the proportion decreased from 59.3% to 53.2% (Table 2).

**Table 2:** Dogs receiving doses of ectoparasitic treatments in veterinary practices in Poland in 2021–2023

	2021	2022	2023
12-week isoxazoline doses/year			
1	1888 (66.8%)	1988 (64.3%)	2130 (62.9%)
2	577 (20.4%)	663 (21.4%)	726 (21.4%)
3	235 (8.3%)	275 (8.9%)	369 (10.9%)
4	101 (3.6%)	146 (4.7%)	147 (4.3%)
5	22 (0.8%)	17 (0.5%)	12 (0.4%)
6	1 (0.0%)	3 (0.1%)	4 (0.1%)
> 6	3 (0.1%)	2 (0.1%)	1 (0.0%
Total	2827 (100%)	3094 (100%)	3389 (100%)
Monthly isoxazoline doses/year			
1	1931 (59.3%)	2614 (56.5%)	3328 (53.2%)
2	604 (18.6%)	811 (17.5%)	1147 (18.3%)
3	282 (8.7%)	466 (10.1%)	652 (10.4%)
4	144 (4.4%)	259 (5.6%)	356 (5.7%)
5	100 (3.1%)	145 (3.1%)	220 (3.5%)
6	84 (2.6%)	116 (2.5%)	195 (3.1%)
> 6	109 (3.3%)	213 (4.6%)	355 (5.7%)
Total	3254 (100%)	4624 (100%)	6253 (100%)
Monthly non-isoxazoline doses/year			
1	628 (77.2%)	564 (76.0%)	451 (75.8%)
2	110 (13.5%)	98 (13.2%)	82 (13.8%)
3	42 (5.2%)	35 (4.7%)	31 (5.2%)
4	14 (1.7%)	20 (2.7%)	15 (2.5%)
5	4 (0.5%)	11 (1.5%)	2 (0.3%)
6	7 (0.9%)	7 (0.9%)	7 (1.2%)
> 6	8 (1.0%)	7 (0.9%)	7 (1.2%)
Total	813 (100%)	742 (100%)	595 (100%)
Collar doses/year			
1	283 (93.4%)	198 (91.7%)	157 (94.6%)
2	17 (5.6%)	16 (7.4%)	7 (4.2%)
3	2 (0.7%)	0 (0.0%)	1 (0.6%)
4	1 (0.3%)	2 (0.9%)	1 0.6%)
5	0 (0.0%)	0 (0.0%)	0 (0.0%)
6	0 (0.0%)	0 (0.0%)	0 (0.0%)
> 6	0 (0.0%)	0 (0.0%)	0 (0.0%)
Total	303 (100.0%)	216 (100.0%)	166 (100.0%)

The prescription rate of more than one dose of products among the analyzed population was low. For the 12-week isoxazolines, the prescription rate of 4 doses that was an approximate equivalent of full-year protection was 3.6% per year, increasing to 4.7% in 2022 and then decreasing to 4.3% in 2023. The rate of administration of more than six doses of monthly isoxazolines (equivalent of at least half a year protection) was 3.3%, 4.6%, and 5.7% in 2021, 2022, and 2023, respectively (Table 2).

Additional analysis was conducted to determine the number of dogs prescribed any product (at least one dose per year) consecutively over a 3-year period. It showed that a relatively small proportion of dogs were consistently prescribed any ectoparasitic treatment at veterinary practices every year. In total, 9.1% (1764 dogs) of the analyzed population (19,351 dogs) received at least one dose of the same product category throughout all study years. Most dogs that received at least one treatment every year were prescribed either 12-week isoxazolines or monthly isoxazolines (Table 3).

**Table 3:** Number of dogs receiving at least one dose of the same product category of ectoparasiticide every year from 45 veterinary practices in Poland during 2021–2023

Product category	Number of dogs	% of population^a^
12-week isoxazoline	876	4.5
Monthly isoxazoline	827	4.3
Monthly non-isoxazoline	46	0.2
Collar	15	0.1
Total	1764	9.1

^a^Percentage calculated among the 19,351 dogs receiving at least one dose of any ectoparasitic product any year during the 3 years of analysis (2021–2023)

### Protection duration

#### Three-year protection

Complete protection against fleas and ticks lasting 3 years equates to approximately 156 weeks of uninterrupted coverage with any ectoparasitic product. The current study reveals that the actual protection durations provided by the products prescribed in veterinary practices in Poland during the analyzed period were significantly shorter than the full 156 weeks (Table 4). Due to differences in efficacy durations against ticks and fleas (some products in the compared categories have varying protection durations as indicated in their SPCs), and given the higher risk of TBDs in Poland, the authors chose to focus on presenting the tick protection durations.

**Table 4:** Protection duration against ticks (weeks ± standard deviation [SD]) of ectoparasiticides prescribed for dogs in veterinary hospitals in Poland for 2021–2023

Product category	Tick average protection
12-week isoxazoline	27.5 ± 25.1^b,c,d^
Monthly isoxazoline	14.2 ± 17.9^a,c,d^
Monthly non-isoxazoline	6.7 ± 7.8^a,b,d^
Collar	42.0 ± 22.7^a,b,c^

Significance of differences among product categories

^a^Significantly different from 12-week isoxazolines (unpaired *t*-test, *P* < 0.05)

^b^Significantly different from monthly isoxazolines (unpaired *t*-test, *P* < 0.05)

^c^Significantly different from monthly non-isoxazolines (unpaired *t*-test, *P* < 0.05)

^d^Significantly different from collars (unpaired *t*-test, *P* < 0.05)

Among the product choices, collars offered the most extended protection, followed by 12-week isoxazolines, monthly isoxazolines, and monthly non-isoxazolines. The average protection prescribed in the form of the 12-week isoxazolines was approximately twice as long as that prescribed in the form of monthly isoxazolines and almost four times as long as that prescribed in the form of monthly non-isoxazolines. Table 4 shows the average length of protection provided for dogs by each category of ectoparasitic products prescribed throughout all 3 years of analysis.

The 19,351 dogs participating in the study should receive 52 weeks of protection per year for 3 years, or 3,018,756 weeks of ectoparasite protection, to ensure full compliance with the year-round recommendation. However, the prescribed product category choices provided 671,794 weeks of protection over the 3 years. Therefore, overall compliance for dogs prescribed ectoparasiticides was 22.3% of the recommended goal for the 3-year study period.

**Fig. 4:**
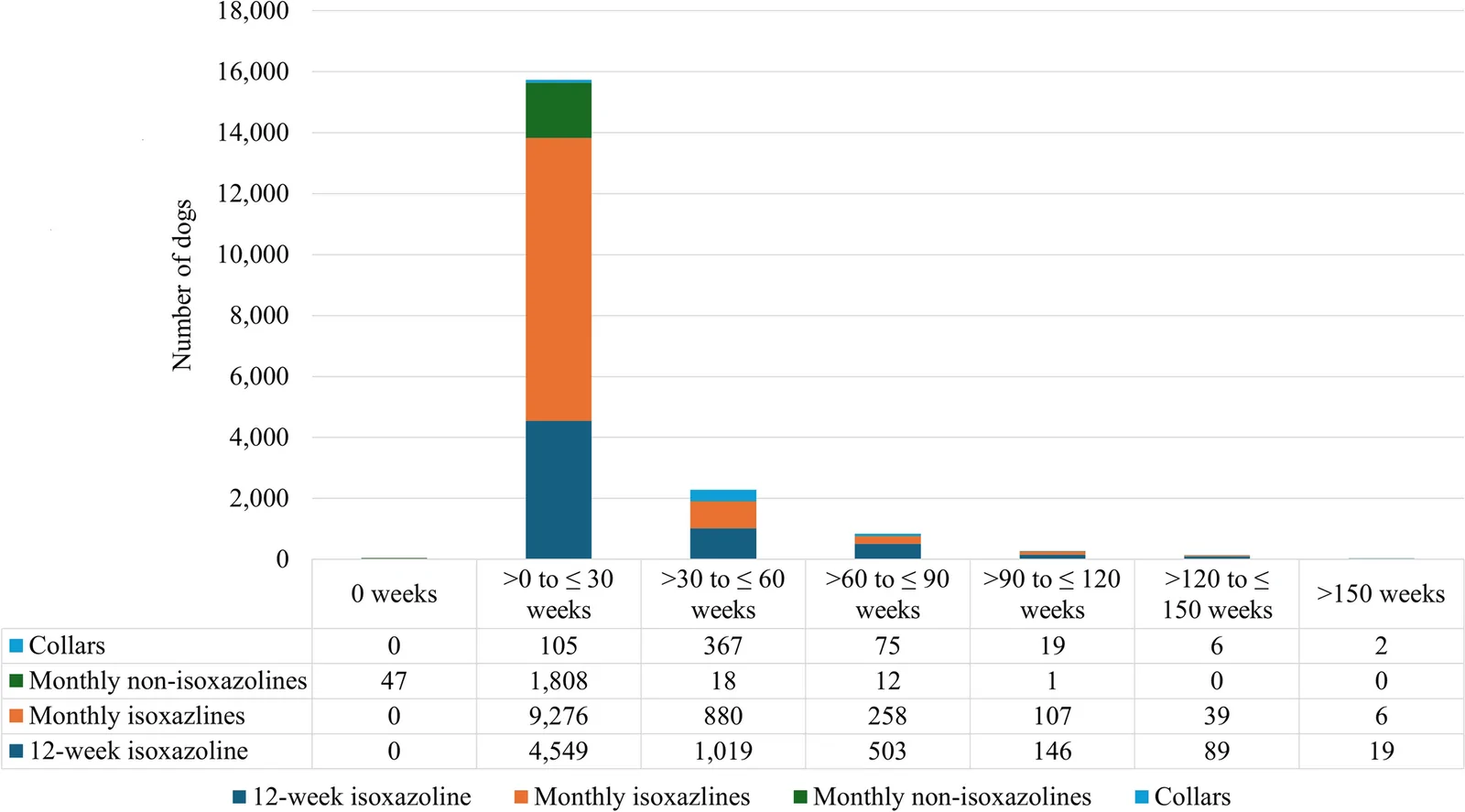
Dogs protected against ticks for different periods of time [weeks] by each product category prescribed in Polish veterinary practices in 2021–2023

Out of the 19,351 dogs in the database, a small group of 27 (0.1%) received year-round protection (≥ 150 weeks) throughout the entire 3-year period analyzed. On the other hand, 47 (0.2%) dogs received no tick protection despite the ectoparasiticide being prescribed, as these products did not have a tick protection indication. Therefore, 99.8% of dogs received protection for less than 156 weeks. Over the 3 years, 15,738 (81.3%) dogs were protected against ticks for up to 30 weeks out of 156 weeks. Furthermore, 2284 (11.8%) of dogs were protected from ticks for 30–60 weeks out of 156 weeks. Similarly, out of an optimal treatment of 156 weeks, 848 (4.4%) were protected for 60–90 weeks, 273 (1.4%) for 90–120 weeks, and 134 (0.7%) for 120–150 weeks (Fig. 4).

#### Yearly protection

The average protection duration across all ectoparasiticide categories differed significantly by year during the analyzed period (Table 5). Collars provide the longest protection (ranging from 34.7 to 35.9 weeks), followed by 12-week isoxazolines (18.2–19.0 weeks), monthly isoxazolines (9.8–11.2 weeks), and non-isoxazoline medications (5.7–6.0 weeks). The length of protection prescribed in the form of a 12-week isoxazoline increased from 2021 to 2023. Although the length of protection provided by monthly isoxazolines increased the most during the 3 years, it remained significantly shorter than that of 12-week isoxazolines and collars.

**Table 5:** Average tick protection duration (weeks) ± standard deviation (SD) prescribed to dogs in Poland per year (2021–2023) for four ectoparasiticide categories

Product category	2021	2022	2023
12-week isoxazoline	18.2 ± 10.8^***,b,c,d^	18.8 ± 10.9^b,c,d^	19.0 ± 10.7^*,b,c,d^
Monthly isoxazoline	9.8 ± 8.5^***,**,a,c,d^	10.5 ± 9.3^*,a,c,d^	11.2 ± 10.0^*,a,c,d^
Monthly non-isoxazoline	5.7 ± 4.5^a,b,d^	5.9 ± 4.7^a,b,d^	6.0 ± 4.8^a,b,d^
Collar	34.7 ± 12.0^a,b,c^	35.9 ± 13.8^a,b,c^	35.2 ± 12.3^a,b,c^

Significance of differences among years within product categories

^*^Different from 2021 within the product category

^**^Different from 2022 within the product category

^***^Different from 2023 within the product category

Significance of differences among product categories within years

^a^Significantly different from 12-week isoxazolines (unpaired *t*-test, *P* < 0.05)

^b^Significantly different from monthly isoxazolines (unpaired *t*-test, *P* < 0.05)

^c^Significantly different from monthly non-isoxazolines (unpaired *t*-test, *P* < 0.05)

^d^Significantly different from collars (unpaired *t*-test, *P* < 0.05)

## Discussion

This study shows that ectoparasitic protection prescribed for dogs in Poland remains substantially below the year-round coverage recommended in current guidelines [1, 6]. Despite the continuous presence of ticks and fleas throughout the year in Central and Eastern Europe, and the well-documented circulation of *I. ricinus* and *D. reticulatus*, the most important vectors of TBDs in Poland [1–5, 10], most dogs analyzed in the present study received only a single ectoparasiticide dose annually across all product categories. Such limited protection leaves dogs exposed for prolonged periods and increases their vulnerability to TBDs, including babesiosis, anaplasmosis, and borreliosis [13–16]. *Dermacentor reticulatus*, the principal vector of *B. canis* in Poland, is widely established, and *I. ricinus* additionally carries *B. burgdorferi* sensu lato and *A. phagocytophilum* [10, 30]. Cross-border movement of dogs, heightened by regional geopolitical instability, further contributes to the introduction of new pathogens [31].

The epidemiological situation is also reinforced by abundant wildlife reservoirs such as wolves, foxes, and ungulates, which maintain endemic transmission of *B. canis*, *A. phagocytophilum*, and *Borrelia* spp. [21–24]. Expansion of *D. reticulatus* into new areas [32–34] and increasing winter activity of ticks [35, 36] compound the year-round exposure risk and justify the recommendation for continuous protection.

While collars are designed to deliver long-lasting ectoparasitic protection for dogs, their effectiveness in everyday use may be frequently compromised. A major issue affecting their performance is collar removal and improper fit, which can lead to gaps in protection if the collar is too loose or otherwise incorrectly positioned on the dog. Additionally, collars can often be removed during bathing, and the bathing process itself may diminish their efficacy. Loss of collars during a dog’ s daily activities [37] also poses a significant challenge, further reducing the period of true effective protection provided by this method. Therefore, the results calculated in the current study must be considered best-case estimates, with the caveat that, in reality, the protection provided by collars is likely to be shorter than these estimates indicate. Unfortunately, the exact calculation of real-life efficacy has not been studied in larger populations.

Systemic isoxazolines, particularly 12-week formulations, offer more consistent protection due to their systemic distribution and reduced sensitivity to environmental conditions and usage particularities [38–42]. Nevertheless, the prescribing patterns observed in the present analysis delivered only 18–19 weeks of protection with 12-week isoxazolines and 9.8–11.2 weeks with monthly products, which is far below the 52 weeks per year required for full protection. Similar disparities between recommended and actual coverage have been reported previously in Europe and the United States [25, 27, 43]. The introduction of isoxazolines in 2014 brought important advantages—high efficacy, prolonged action, and minimal interference with bathing—yet even these products must be administered multiple times annually to maintain continuous protection [25, 27, 38–46].

Owner behavior appears central to low adherence. Many owners purchase ectoparasiticides only once per year and do not return for refills, consistent with findings from Spain and the United States [25–27]. Potential contributors include low awareness of year-round risk, belief that protection is needed only in warm months, financial limitations, or a general tendency to defer preventive care. Notably, a rising number of prescriptions over the 3-year study period did not translate into longer protection, underscoring the fact that increasing product dispensing alone does not improve compliance, which requires targeted communication as well.

The authors acknowledge several limitations inherent in the present analysis, which is based exclusively on veterinary practice prescription records to assess ectoparasitic protection. One key limitation is that the study captures data from a single source; it is possible that the dogs included in the analysis received ectoparasitic protection from additional veterinary practices or through other channels where such products are available. However, the analysis of prescription records from veterinary practices is an approach that has been employed in several previous studies [25–27]. Alternative methodologies have not been widely adopted in scientific publications [6]. While market studies investigating owner behavior tend to reflect intent to use ectoparasiticides, they do not necessarily reflect actual usage patterns. Therefore, prescription record analysis likely provides the most accurate reflection of real-world use among the currently available methodologies. Furthermore, this method enables the examination of a large volume of records, offering a broader perspective than questionnaire-based studies, which are inherently limited by smaller sample sizes and dependent on pet owner memory.

Given the substantial burden and clinical severity of TBDs in Poland [13, 16, 18], improving adherence to prevention recommendations should be a priority for veterinary practices. Strengthening communication—emphasizing the epidemiology of ticks in Poland, persistent exposure risk, and the practical benefits of long-acting systemic formulations—may help to close the compliance gap. From a broader public health perspective, more consistent use of long-acting systemic ectoparasiticides could reduce TBD incidence in dogs and mitigate potential zoonotic spillover involving *Borrelia*, *Anaplasma*, and other pathogens.

Finally, solutions that reduce reliance on owner adherence warrant attention. Although 12-week isoxazolines already offer better adherence than short-acting (monthly) products, they still require multiple annual doses. Future single-dose, year-long formulations may provide the most effective means of achieving sustained protection that aligns with the continuous ectoparasite and TBD pressure observed in Poland.

## Conclusions

The duration of ectoparasite protection prescribed by Polish veterinary practices for dogs is significantly shorter than the recommended 12 months per year for over 99% of dogs. More than 90% of dogs received six or fewer doses of ectoparasiticide annually over the 3 years covered by the study. Long-acting products offer longer flea and tick protection, improving compliance, as most dogs are prescribed only one ectoparasiticide dose per year at veterinary practices. Year-to-year treatment decisions are inconsistent. Fewer than 10% of dogs receive the same treatment for three consecutive years, still missing the full-time coverage. Lack of follow-through leaves dogs vulnerable to ectoparasites and vector-borne diseases. Pet owners’ purchasing behaviors suggest that the ideal ectoparasiticide should provide 12 months of protection from a single dose, ensuring year-round control of fleas and ticks.

## Supplementary Information

Supplementary material 1.

Supplementary material 2.

## Data Availability

Data supporting the main conclusions of this study are included in the manuscript.

## Abbreviations

TBD: Tick-borne disease
SPC: Summary of product characteristics
