# Supplementary material for: Ectoparasitic prevention compliance in dogs based on prescription records from veterinary practices in Poland
Source: Parasit Vectors. 2026 Jul 22;19:456. doi: 10.1186/s13071-026-07374-5 (PMC13682044; doi:10.1186/s13071-026-07374-5)
Supplement: Supplementary file 1 [file 13071_2026_7374_MOESM1_ESM.docx]

**Table S1.** Number of ectoparasiticide doses and dogs prescribed medications from the categories (percentage of the year-over-year change).

| **Category** | Doses 2021 | Dogs 2021 | Doses 2022 | Dogs 2022 | Doses 2023 | Dogs 2023 |
| --- | --- | --- | --- | --- | --- | --- |
| 12-week isoxazoline | 4,296 | 2,827 | 4,842 (+13%) | 3,094 (+9%) | 5,369 (+11%) | 3,389 (+10%) |
| Monthly isoxazoline | 6,464 | 3,254 | 9,895 (+53%) | 4,624 (+42%) | 14,307 (+45%) | 6,253 (+35%) |
| Monthly-non isoxazoline | 1,165 | 813 | 1097 (-6%) | 742 (-9%) | 879 (-20%) | 595 (-20%) |
| Collar | 327 | 303 | 238 (-27%) | 216 (-29%) | 178 (-25%) | 166 (-23%) |
| Total | 12,252 | 7,197 | 16,072 (+31%) | 8,676 (+21%) | 20,733 (+29%) | 10,403 (+20%) |
