# Supplementary material for: Ectoparasitic prevention compliance in dogs based on prescription records from veterinary practices in Poland
Source: Parasit Vectors. 2026 Jul 22;19:456. doi: 10.1186/s13071-026-07374-5 (PMC13682044; doi:10.1186/s13071-026-07374-5)
Supplement: Supplementary file 2 [file 13071_2026_7374_MOESM2_ESM.docx]

**Table S2.** Average flea protection duration (weeks) ± SD provided to dogs in Poland per year (2021-2023) for four ectoparasiticide categories.

| **Product category** | **2021** | **2022** | **2023** |
| --- | --- | --- | --- |
| 12-week isoxazoline | 18.2±10.8^***,b,c,d^ | 18.8±10.9^b,c,d^ | 19.0±10.7^*,b,c,d^ |
| Monthly isoxazoline | 9.9±8.5^**,a,c,d^ | 10.6±9.5^*,***,a,c,d^ | 11.4±10.2^**,a,c,d^ |
| Monthly non-isoxazoline | 7.4±6.9^***, a,b,d^ | 7.4±6.7^***, a,b,d^ | 6.5±5.3^*,**,a b,d^ |
| Collar | 34.7±12.0^a,b,c^ | 35.9±13.8^a,b,c^ | 35.2±12.3^a,b,c^ |

All results within a column are significantly different (T-test, p<0.05)

* - different from 2021

** - different from 2022

***- different from 2023

Significance of differences among product categories within years

a - significantly different from 12-week isoxazoline (unpaired t-test, p<0.05)

b - significantly different from monthly isoxazolines (unpaired t-test, p<0.05)

c -significantly different from monthly non-isoxazolines (unpaired t-test, p<0.05)

d -significantly different from collars (unpaired t-test, p<0.05)
